# Tissue inhibitor of metalloproteinase 1 promotes ferroptosis and suppresses prostate cancer metastasis

**DOI:** 10.1016/j.jbc.2025.108473

**Published:** 2025-04-02

**Authors:** Yuliang Rao, Qi Pan, Siyu Liu, Shunheng Yao, Lei Li, Jianyan Yan, Lifen Chen, Li Xu, Han Yan, Aicui Ma, Fen Wang, Xiaoyan Mao, Zhonghui Wang, Junfang Zhang, Jun Guo, Zuyue Sun

**Affiliations:** 1 School of Pharmacy, School of Basic Medical Sciences, Fudan University, Shanghai, China; 2 National Evaluation Centre for the Toxicology of Fertility Regulating Drug, Shanghai Institute for Bio-medical and Pharmaceutical Technologies (SIBPT), Shanghai, China; 3 NHC Key Laboratory of Reproduction Regulation, Shanghai, China; 4 Shanghai Engineering Research Center of Reproductive Health Drug and Devices, Shanghai, China

**Keywords:** prostate cancer, TIMP1, ferroptosis, GPX4, metastasis

## Abstract

Tissue inhibitor of metalloproteinase 1 (TIMP1) has been implicated in prostate cancer (PCa) metastasis. In this study, PC-3M-2B4 cells with TIMP1 knockdown (PC-3M-2B4-shTIMP1) or overexpression (PC-3M-2B4-TIMP1) were generated, and an inverse correlation was found between TIMP1 expression and cell migration and invasion, which was confirmed *in vitro* and *in vivo*. Differential TIMP1 expression was accompanied by variations in the expression of the ferroptosis-related proteins, glutathione peroxidase 4 (GPX4), transferrin receptor, transferrin, glutamine cysteine ligase catalytic subunit, and glutamine cysteine ligase modifier subunit. In comparison with TIMP1-overexpressing cells, TIMP1-knockdown cells demonstrated a 12.3% decrease in Fe^2+^ concentration after erastin treatment, a 37.8% reduction in malondialdehyde levels, an 113.7% increase in GPX4 expression, and a 78.9% rise in the GSH–GSSG ratio. Our findings indicate that TIMP1 overexpression promotes ferroptosis by modulating critical markers, such as GPX4 and transferrin receptor, thereby significantly reducing metastatic potential in PCa cells. Our results highlight the role of TIMP1 in regulating ferroptosis pathways, which are crucial for tumor progression, and exposes a potential therapeutic target for PCa management.

Prostate cancer (PCa) remains one of the most prevalent cancers among men worldwide, accounting for approximately 14% of all newly diagnosed cancers in males and ranking as the second leading cause of cancer-related deaths in this demographic (1, 2). While advances in diagnostic techniques and treatment strategies have significantly improved the 5-year survival rate for localized PCa to nearly 100%, metastatic PCa continues to pose a significant clinical challenge, contributing disproportionately to cancer mortality (3). Understanding the molecular mechanisms driving PCa metastasis is therefore critical for developing targeted therapeutic interventions.

Tissue inhibitors of metalloproteinases (TIMPs), particularly TIMP1, play multifaceted roles in cancer biology. Traditionally recognized as inhibitors of matrix metalloproteinases (MMPs), TIMPs regulate extracellular matrix (ECM) remodeling and influence processes, such as cell proliferation, apoptosis, and angiogenesis (4, 5). Elevated TIMP1 expression has been reported in multiple cancers, including lung, breast, and PCas, often correlating with aggressive tumor behavior and poor prognosis (6). However, its role in PCa remains complex, with some studies suggesting a protective effect against metastasis through suppression of MMP activity (7, 8).

Recent research has identified ferroptosis, a regulated form of iron-dependent cell death characterized by lipid peroxidation, as a critical pathway in cancer progression and therapy (9, 10). Tumor cells, including those in PCa, exhibit heightened metabolic and oxidative activity, making them particularly susceptible to ferroptosis inducers (FINs) (11, 12). Notably, ferroptosis has been implicated in immune modulation, tumor microenvironment remodeling, and therapy resistance, positioning it as a promising target for cancer treatment (13). This indicates that cancer cells may be more susceptible to FINs because of their elevated metabolic activity, elevated reactive oxygen species levels, and iron requirements (14, 15, 16, 17, 18). It is noteworthy that mesenchymal and dedifferentiated cancer cells, which are typically resistant to apoptosis and conventional therapies, are particularly susceptible to ferroptosis (19, 20, 21, 22). It can thus be concluded that FINs represent a promising avenue for cancer treatment (23, 24). Despite its potential, the relationship between TIMP1 and ferroptosis in the context of PCa metastasis remains largely unexplored.

In this study, we investigate the dual role of TIMP1 as a modulator of ferroptosis and a regulator of metastatic potential in PCa. By leveraging cellular, molecular, and *in vivo* approaches, we examine how TIMP1 expression influences key ferroptosis markers, including glutathione peroxidase 4 (GPX4) and transferrin receptor (TFRC), and its impact on PCa progression. Our findings provide novel insights into the interplay between TIMP1 and ferroptosis, highlighting its potential as a therapeutic target for advanced PCa.

## Results

### TIMP1 expression negatively correlates with PCa cell proliferation, migration, and invasion *in vitro*

**Figure 1:**
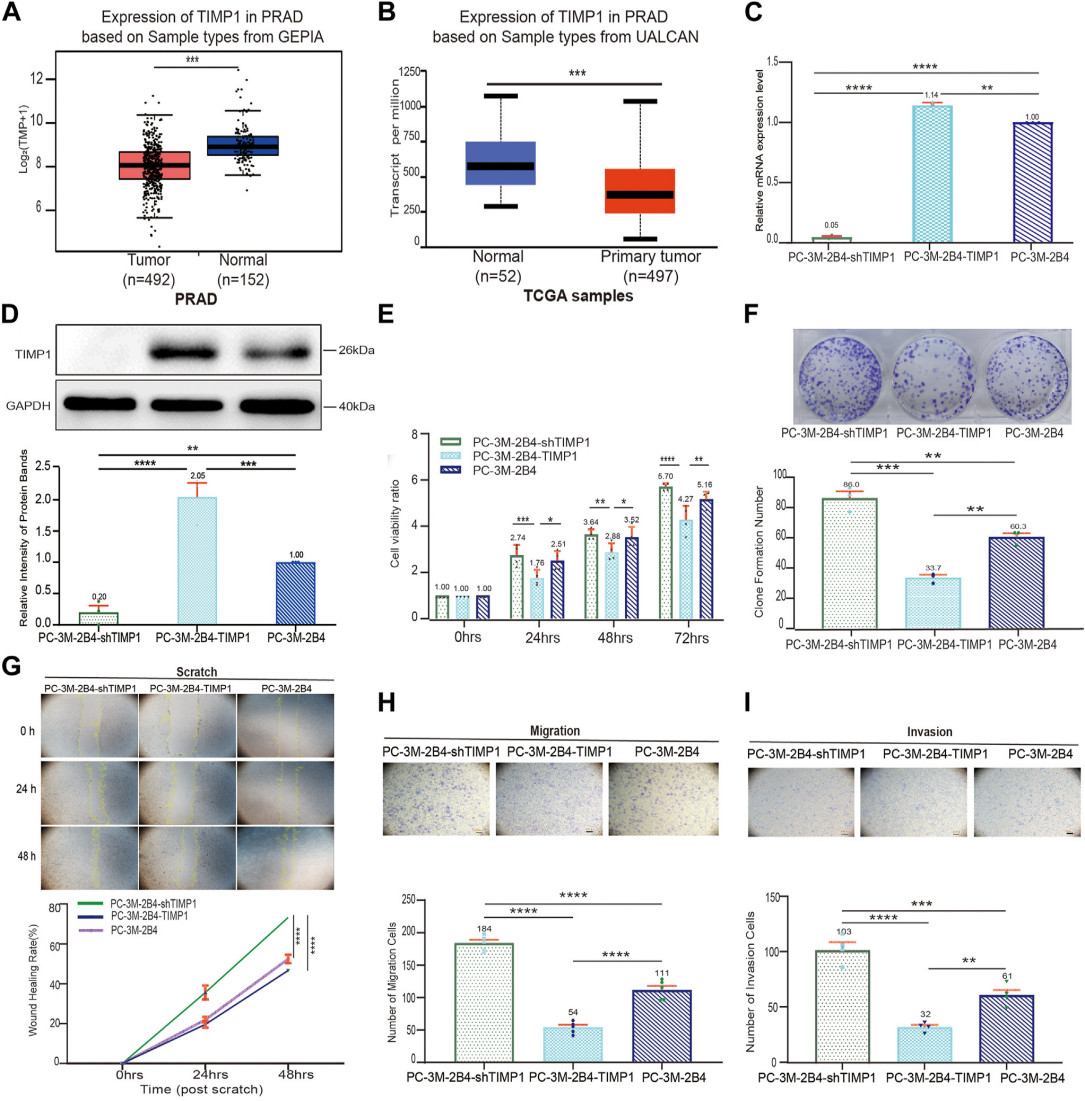
**TIMP1 expression negatively correlates with prostate cancer cell proliferation, migration, and invasion *in vitro*.***A* and *B*, TIMP1 mRNA in primary prostate adenocarcinoma tissues and adjacent normal tissues from the GEPIA and UALCAN databases. *C* and *D*, TIMP1 expression in PC-3M-2B4-shTIMP1, PC-3M-2B4-TIMP1, and PC-3M-2B4 cells. *E*, cell proliferation and viability ratios of PC-3M-2B4-TIMP1, PC-3M-2B4-shTIMP1, and PC-3M-2B4 at 24, 48, and 72 h relative to 0 h (n = 4). *F*, *left*, images of cell clone formation; *right*, cell clone formation bar chart (n = 3). *G*, representative images of wound healing assays. Initial scratch widths may vary slightly because of experimental variability, but wound closure rates were calculated based on relative changes over time. Scale bar represents 200 μm. *Yellow line*: boundary plot of wound healing fitted by ImageJ; wound healing rate = (distance of 0 h scratch - distance of scratch at each time point)/distance of 0 h scratch × 100% (n = 4). *H*, *upper*, images of cell migration (200x magnification); *lower*, cell migration graph (n = 5). *I*, *upper*, images of cell invasion (200×); cell invasion graph (n = 4). ∗*p <* 0.05, ∗∗*p <* 0.01, ∗∗∗*p <* 0.001, and ∗∗∗∗*p <* 0.0001. All data are shown as the mean ± SD from n = 3∼5 biological replicates. TIMP1, tissue inhibitor of metalloproteinase 1.

Analysis of prostate adenocarcinoma samples from the GEPIA and UALCAN databases revealed significantly lower TIMP1 mRNA expression in tumor tissues compared with adjacent normal tissues (*p <* 0.001) (Fig. 1, *A* and *B*). This differential expression prompted the generation of stable PCa cell lines with TIMP1 knockdown (PC-3M-2B4-shTIMP1) or overexpression (PC-3M-2B4-TIMP1) (Fig. 1, *C* and *D*). TIMP1 knockdown cells exhibited a 1.33-fold increase in proliferation (measured by Cell Counting Kit-8 [CCK-8] assay at 72 h) compared with TIMP1-overexpressing cells (*p <* 0.05) (Fig. 1*E*). Consistent with these findings, colony formation was significantly reduced in PC-3M-2B4-TIMP1 cells, showing a 61% reduction compared with PC-3M-2B4-shTIMP1 cells (*p <* 0.01) (Fig. 1*F*).

Wound healing assays further supported these findings. The wound closure rate was 1.57-fold higher in PC-3M-2B4-shTIMP1 cells than in PC-3M-2B4-TIMP1 cells (*p <* 0.05) (Fig. 1*G*). Notably, TIMP1 overexpression did not significantly alter migration compared with parental PC-3M-2B4 cells, suggesting that TIMP1 knockdown, rather than overexpression, drives enhanced migratory behavior (Fig. 1*G*). Furthermore, migration and invasion assays showed that PC-3M-2B4-shTIMP1 cells had significantly higher migration (3.41-fold, *p <* 0.001) and invasion (3.22-fold, *p <* 0.001) rates compared with PC-3M-2B4-TIMP1 cells (Fig. 1, *H* and *I*). Collectively, these findings indicate that TIMP1 expression suppresses proliferation, migration, and invasion in PCa cells, highlighting its role as a negative regulator of metastatic behaviors.

### TIMP1 reduces tumor growth and metastasis *in vivo*

**Figure 2:**
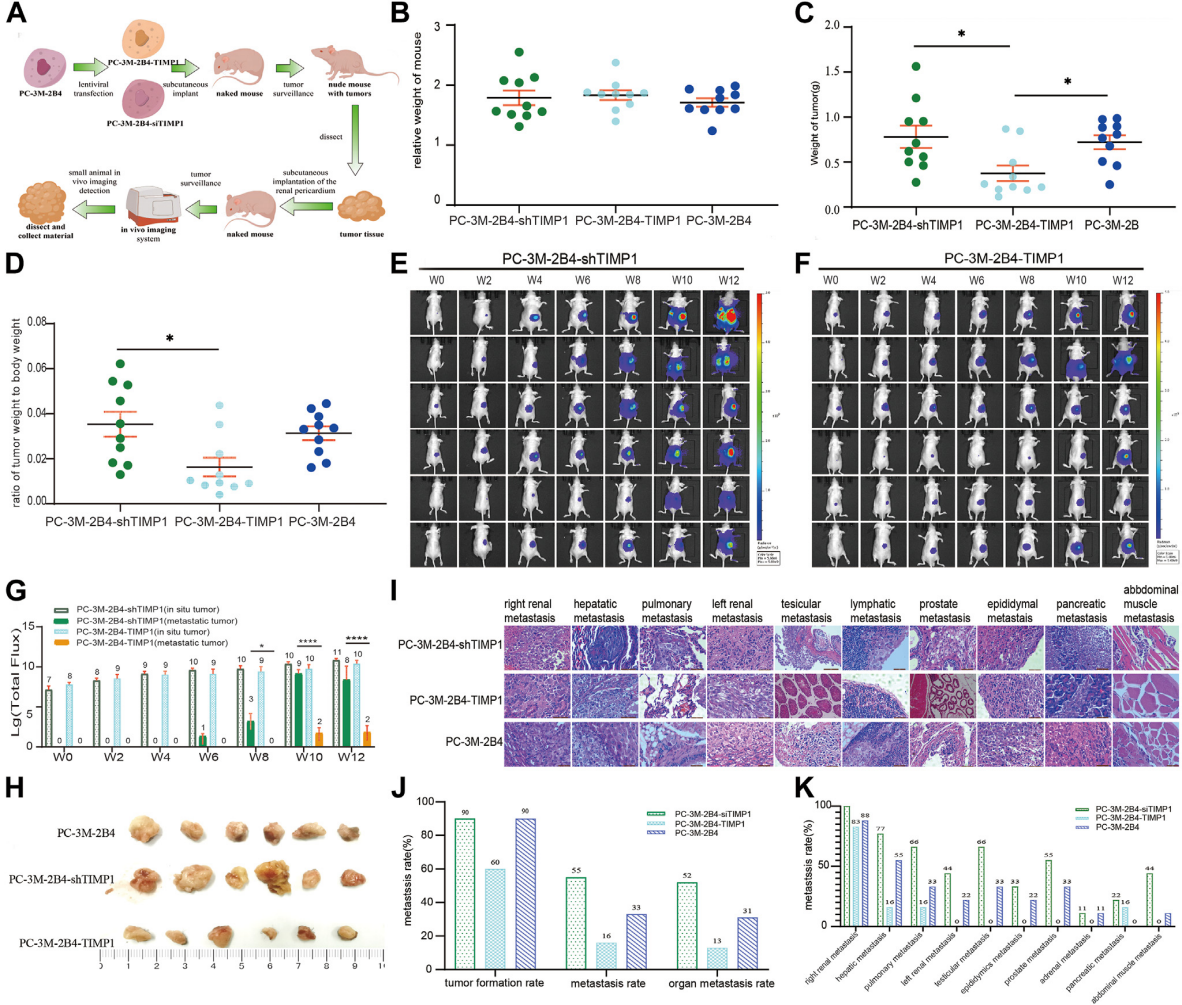
**TIMP1 reduces tumor growth and metastasis *in vivo*.***A*, flowchart of subperitoneal graft tumors in nude mice. *B*, ratio of tumor-bearing body weight to original body weight in nude mice at week 12 (n = 10). *C*, nude mouse tumor weight (n = 10). *D*, tumor weight:body weight ratio (n = 10). *E*, bioluminescence images of PC-3M-2B4-shTIMP1 tumors. *Right hand bar* shows the logarithm of fluorescence intensity. *F*, bioluminescence images of PC-3M-2B4-TIMP1 tumors. *G*, logarithm of fluorescence intensity of tumor graft sites and metastatic sites (n = 10). *H*, morphology of subperitoneal graft tumors. *I*, representative HE staining results of primary and metastatic tumors. *J*, tumor formation, metastasis, and organ metastasis rates of transplanted tumors. *K*, metastasis rates of chief organ sites. Organ metastasis rate: percentage of metastases detected by pathology/number of tumor-bearing mice; total organ metastasis rate: percentage of the total number of organs with metastasis out of *right* kidney, liver, lung, *left* kidney, testis, epididymis, prostate, adrenal gland, pancreas, and abdominal wall muscle/total number of organs. ∗*p <* 0.05, ∗∗*p <* 0.01, ∗∗∗*p <* 0.001, and ∗∗∗∗*p <* 0.0001. TIMP1, tissue inhibitor of metalloproteinase 1.

To evaluate the role of TIMP1 *in vivo*, tumor-bearing nude mice were generated using subperitoneal transplantation of PC-3M-2B4-shTIMP1, PC-3M-2B4-TIMP1, or PC-3M-2B4 control cells (Fig. 2*A*). Tumor in the PC-3M-2B4-TIMP1 group exhibited a 58% reduction in weight compared with the PC-3M-2B4-shTIMP1 group (*p <* 0.01) (Fig. 2, *B* and *C*), accompanied by a significant decrease in the tumor-to-body weight ratio (Fig. 2*D*). Metastatic potential was assessed using *in vivo* imaging and histological examination (Fig. 2, *E*–*G*). At 6 weeks, bioluminescence signals indicated metastasis in 55% of PC-3M-2B4-shTIMP1 mice (Fig. 2*E*), compared with only 16% of PC-3M-2B4-TIMP1 mice (Fig. 2*F*). Histological analysis confirmed these findings, showing a 3.43-fold higher total metastasis rate and a fourfold increase in organ metastasis in PC-3M-2B4-shTIMP1 mice compared with PC-3M-2B4-TIMP1 mice (Fig. 2, *H*–*J*). Organ-specific metastases were primarily observed in the kidney, liver, lung, and pancreas (Fig. 2, *I* and *K*). Collectively, these results demonstrate that TIMP1 overexpression significantly reduces tumorigenicity and metastatic potential in PCa, underscoring its role as a key regulator of tumor progression (Table S2).

### TIMP1 modulates ferroptosis pathways in PCa

**Figure 3:**
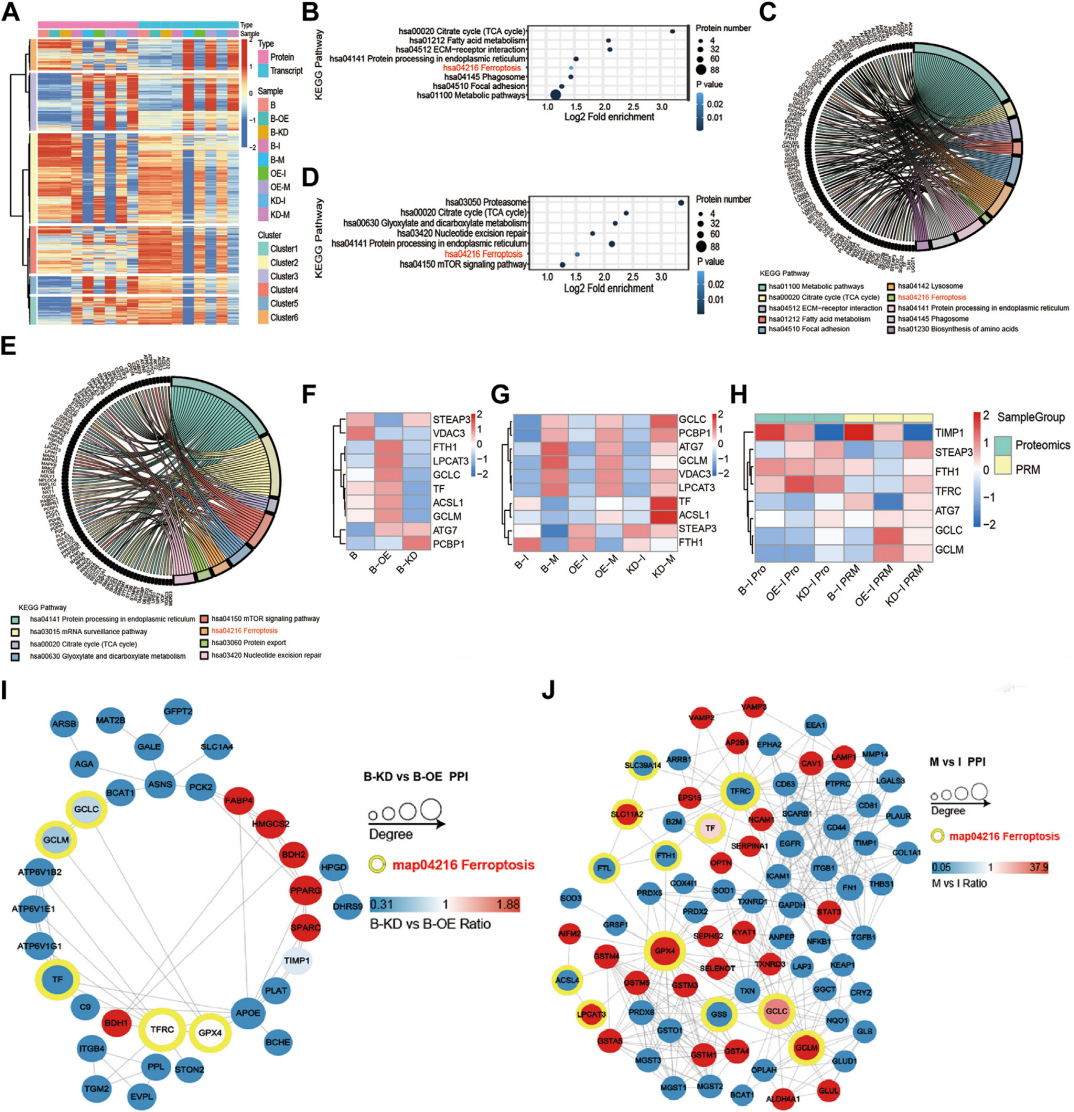
**Multiomics profiling of TIMP1-regulated ferroptosis pathways in prostate cancer.***A*, heatmap of differentially expressed proteins/transcripts across cell lines (PC-3M-2B4), primary tumors (I), and metastatic tumors (M) in athymic mice. *B*, KEGG pathway enrichment (cluster 1): bubble plot highlights ferroptosis pathway. *C*, chord diagram visualizes gene-pathway associations for cluster 1. *D*, KEGG pathway enrichment (cluster 3): bubble plot emphasizes ferroptosis pathway. *E*, chord diagram for cluster 3 pathways. *F*, heatmap of ferroptosis-related protein expression in TIMP1-overexpressing (OE) *versus* knockdown (KD) cells. *G*, heatmap of ferroptosis-related proteins in primary (I) *versus* metastatic (M) tumors. *H*, PRM validation of differentially expressed proteins in primary tumors. *I*, protein–protein interaction (PPI) network in TIMP1-KD *versus* TIMP1-OE cells. *J*, PPI network in metastatic *versus* primary tumors. Heatmaps display mean log2 fold changes across three biological replicates. Statistical significance was determined using FDR correction (∗FDR *<*0.1, ∗*p <* 0.05). n = 3. Key: B, PC-3M-2B4; I, primary tumor; M, metastatic tumor. FDR, false discovery rate; KEGG, Kyoto Encyclopedia of Genes and Genomes; PRM, parallel reaction monitoring; TIMP1, tissue inhibitor of metalloproteinase 1.

**Figure 4:**
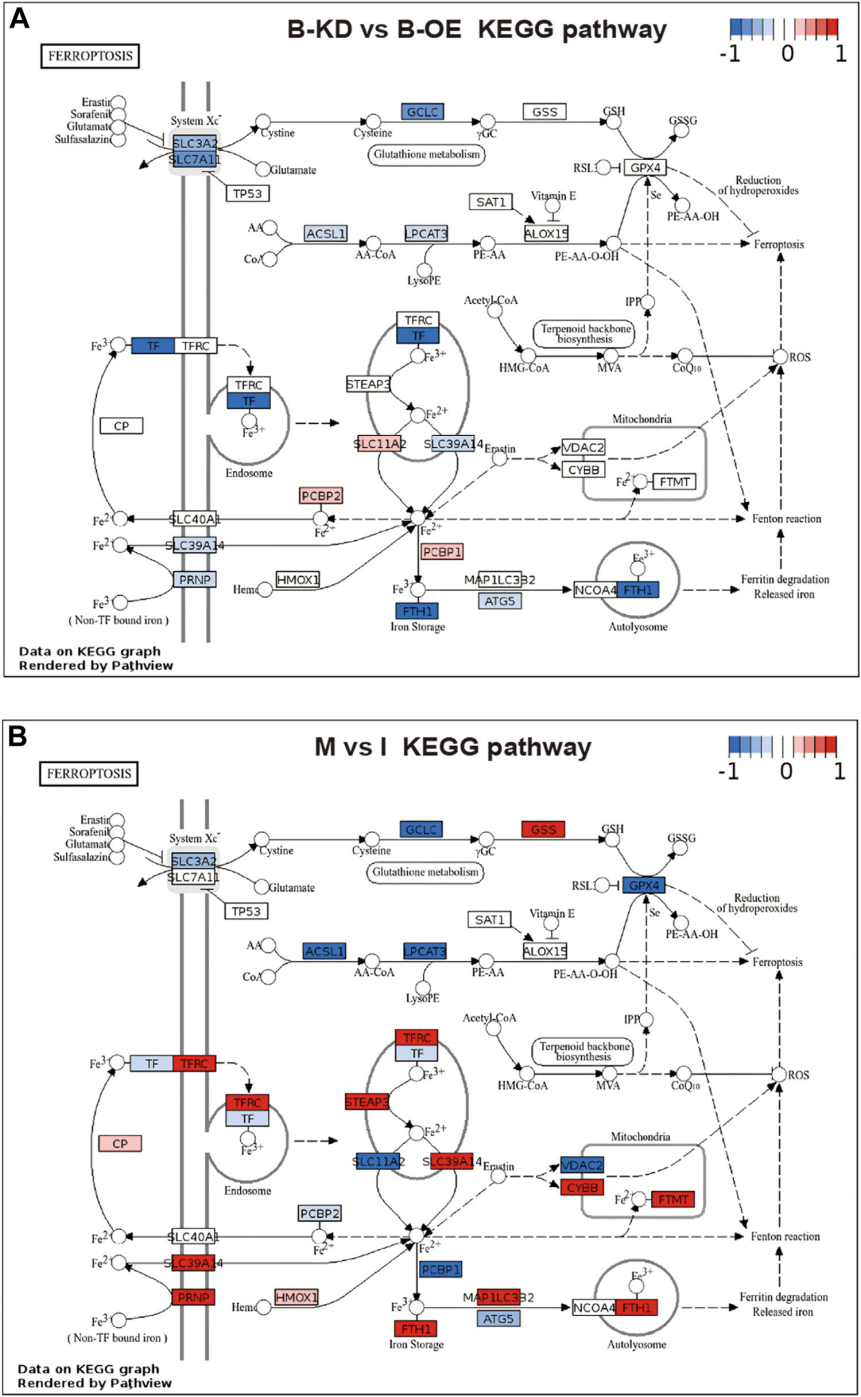
**TIMP1 modulates ferroptosis pathways in cellular and tumor contexts.***A*, ferroptosis pathway in TIMP1-knockdown (KD) *versus* TIMP1-overexpressing (OE) cells. *B*, ferroptosis pathway alterations in metastatic (M) *versus* primary (I) tumors. TIMP1, tissue inhibitor of metalloproteinase 1.

Proteomic and transcriptomic analyses were conducted to explore the molecular mechanisms underlying the impact of TIMP1 on metastasis. Heatmap clustering of differentially expressed proteins in TIMP1-modified cells, as well as *in situ* and metastatic tumor samples from nude mice, revealed significant concordance in clusters 1 and 3 (Fig. 3*A*). Cluster 1 was enriched for several major pathways (Fig. 3*B*), with the ferroptosis pathway (hsa04216) emerging as a key player, as illustrated in the corresponding chord diagram (Fig. 3*C*). Similarly, cluster 3 also highlighted the ferroptosis pathway as one of the most significant pathways (Fig. 3, *D* and *E*). Heatmap analysis of differentially expressed proteins revealed that key ferroptosis-associated proteins—including transferrin (TF), glutamine cysteine ligase catalytic subunit (GCLC), and glutamine cysteine ligase modifier subunit (GCLM)—exhibited consistent expression patterns across TIMP1-modulated cells, primary tumors, and metastatic tumors. These trends aligned with distinct biological phenotypes, suggesting that TIMP1 expression levels drive functional changes closely linked to ferroptosis-related protein dynamics (Fig. 3, *F* and *G*). Parallel reaction monitoring analysis further validated these results (Fig. 3*H*). *In vivo* tumor analysis corroborated these findings, revealing elevated expression of GPX4, GCLC, and GCLM in TIMP1-knockdown tumors. Protein–protein interaction network analysis revealed strong interactions among ferroptosis proteins, including GPX4, TFRC, and GCLC, suggesting a central regulatory role for TIMP1 in ferroptosis (Fig. 3, *I* and *J*). Further analysis of ferroptosis pathway proteins, including TF, GCLC, and GCLM, demonstrated differential involvement in TIMP1-overexpressing and TIMP1-knockout cells and tumor tissues (Fig. 4, *A* and *B*). Collectively, these findings indicate that the ferroptosis pathway, particularly through key proteins, such as TF, TFRC, GPX4, GCLC, and GCLM, plays a critical role in PCa metastasis and is modulated by TIMP1.

### TIMP1 modulates cellular responses to ferroptosis inducers and inhibitors

**Figure 5:**
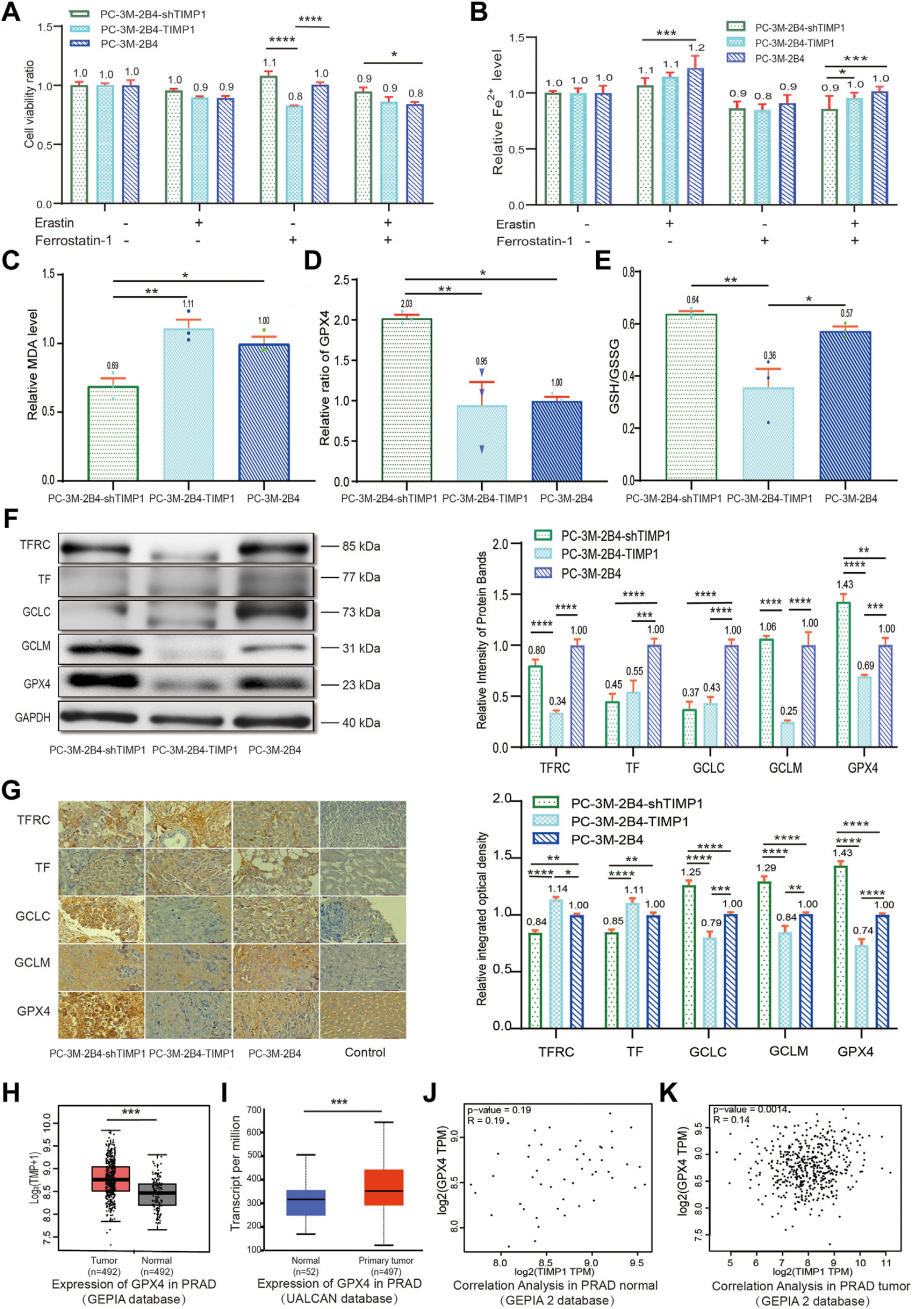
**TIMP1 modulates ferroptosis sensitivity in prostate cancer (PCa) cells.***A*, cell viability after erastin/ferrostatin-1 treatment. *B*, erastin-induced Fe^2+^ accumulation in PC-3M-2B4 (parental), PC-3M-2B4-shTIMP1 (knockdown), and PC-3M-2B4-TIMP1 (overexpression) cells. While TIMP1 knockdown suppresses Fe^2+^ accumulation, TIMP1 overexpression limits Fe^2+^ increase because of altered iron metabolism (*e.g.*, TFRC downregulation). Data represent mean ± SEM; *p <* 0.05 *versus* parental, #*p <* 0.05 *versus* shTIMP1. *C*, MDA levels, a marker of lipid peroxidation. *D* and *E*, GPX4 expression and GSH/GSSG ratio. *F*, WB analysis of ferroptosis proteins in cells with TIMP1 differential expression. *G*, IHC staining and relative integrated optical density (IOD) analysis of TFRC, TF, GCLC, GCLM, and GPX4 in tumor tissue. *F* and *G*, mRNA and protein levels of ferroptosis-related markers. Note the discordance for TFRC, likely reflecting post-transcriptional regulation (see Discussion section). Data represent mean ± SEM; *p <* 0.05 *versus* parental. *H* and *I*, GPX4 mRNA in PCa and adjacent normal tissues from the GEPIA (*H*) and UALCAN (*I*) databases. *J*, correlation analysis of TIMP1 and GPX4 in PRAD adjacent control tissue (GEPIA 2 database). *K*, correlation analysis of TIMP1 and GPX4 in PRAD tumor tissue (GEPIA 2 database). ∗*p <* 0.05, ∗∗*p <* 0.01, ∗∗∗*p <* 0.001, and ∗∗∗∗*p <* 0.0001. All data are shown as the mean ± SD from n = 3 ∼ 4 biological replicates. GCLC, glutamine cysteine ligase catalytic subunit; GCLM, glutamine cysteine ligase modifier subunit; GPX4, glutathione peroxidase 4; IHC, immunohistochemical; MDA, malondialdehyde; PRAD, prostate adenocarcinoma; TF, transferrin; TFRC, transferrin receptor; TIMP1, tissue inhibitor of metalloproteinase 1; WB, Western blot.

To assess the role of TIMP1 in ferroptosis directly, cells were treated with the FIN erastin or inhibitor ferrostatin-1. Ferrostatin-1 treatment significantly elevated proliferation in TIMP1-knockdown cells, increasing viability by 1.3-fold relative to TIMP1-overexpressing cells, and reducing viability in the TIMP-1-overexpressing cells by 0.2-fold compared with PC-3M-2B4 cells (Fig. 5*A*). PC-3M-2B4TIMP1-overexpressing cells demonstrated heightened sensitivity to ferroptosis induction, displaying as evidenced by a 12.3% reduction in Fe^2+^ concentration compared with TIMP1-knockdown cells (PC-3M-2B4-shTIMP1) after erastin treatment (Fig. 5*B*). TIMP1 knockdown further reduced malondialdehyde (MDA) content by 37.8% (Fig. 5*C*), elevated GPX4 expression by 113.7% (Fig. 5*D*), and increased the GSH–GSSG ratio by 78.9% (Fig. 5*E*) compared with TIMP1-overexpressing cells (*p <* 0.001). We conclude that overexpression of TIMP1 enhances ferroptosis-related markers, including MDA (a lipid peroxidation product), and decreases antioxidant capacity (lower GSH–GSSG ratio), as demonstrated in Figure 5, *C*–*E*. Western blot (Fig. 5*F*) and immunohistochemical (IHC) (Fig. 5*G*) analyses further supported these findings, showing differential expression of ferroptosis markers, such as GPX4, TFRC, and GCLC in TIMP1-knockdown and -overexpressing cells. Notably, higher GPX4 levels in TIMP1-knockdown cells aligned with reduced ferroptosis susceptibility, underscoring the role of TIMP1-knockdown expression in suppressing oxidative stress–induced cell death. TIMP1 overexpression reduces GCLM (as per Western blotting); this could limit metastasis by inducing ferroptosis in disseminated cells. Conversely, TIMP1 knockdown increases GCLM, potentially enhancing metastatic fitness *via* GSH-mediated antioxidant protection (Fig. 5, *F* and *G*).

## Discussion

Our study demonstrates that TIMP1 overexpression enhances ferroptosis in PCa by promoting lipid peroxidation and depleting antioxidant defenses, as evidenced by elevated MDA levels and reduced GSH/GSSG ratios (Fig. 5, *C*–*E*). Our data reveal that TIMP1 suppresses GCLM expression, depleting GSH and sensitizing cells to ferroptosis. This mechanism may underpin the antimetastatic effects of TIMP1, as GSH loss compromises antioxidant defenses critical for circulating tumor cell survival. Conversely, TIMP1 knockdown elevates GCLM, potentially fostering metastasis through GSH-mediated stress resistance. These findings reveal a novel role for TIMP1 in regulating redox homeostasis and ferroptosis sensitivity, extending its established functions in ECM remodeling and metastasis suppression (7, 25). The relative contribution of these pathways may depend on tumor stage, microenvironmental stressors, and TIMP1 expression levels (8, 26, 27).

The proferroptotic effect of TIMP1 is mechanistically linked to oxidative stress pathways. Elevated MDA levels in TIMP1-overexpressing cells reflect iron-dependent lipid peroxidation, a hallmark of ferroptosis (13), whereas diminished GSH–GSSG ratios indicate compromised antioxidant capacity (28). Overexpression of TIMP1 downregulates GPX4 compared with TIMP1-knockdown cells, though this effect is less pronounced relative to untransfected PC-3M-2B4 cells. This suggests that the regulatory impact of TIMP1 on GPX4 is context dependent and may be more relevant in settings of altered TIMP1 expression (8, 29, 30, 31). Notably, TIMP1 knockdown led to upregulation of GPX4 (Fig. 5*D*), a pivotal antioxidant enzyme whose loss is central to ferroptosis execution (14). This suggests that TIMP1 suppresses GPX4 transcription or stability, tipping the balance toward oxidative damage (8, 29). The regulation of ferroptosis-related proteins by TIMP1 operates through transcriptional and iron-dependent mechanisms. Elevated TIMP1 suppresses GPX4, likely *via* Nrf2 inhibition (23, 32, 33, 34, 35), while concurrently altering iron metabolism to restrict labile Fe^2+^ pools. The paradoxical increase in TFRC protein—despite reduced mRNA—highlights the role of TIMP1 in iron homeostasis, where Fe^2+^ depletion activates iron regulatory protein–mediated stabilization of TFRC transcripts (19). Furthermore, the interaction of TIMP1 with NF-κB and mitogen-activated protein kinase pathways (31) may amplify oxidative stress by repressing antioxidant defenses. These multilayered effects position TIMP1 as a central node coordinating iron metabolism and redox balance in PCa.

Furthermore, TIMP1 expression led to a reduction in intracellular Fe^2+^ (Fig. 5*B*), resulting in an iron starvation mimicking effect that was followed by the triggering of iron regulatory protein–mediated TFRC translation, thus restoring iron homeostasis (22, 36, 37). This finding highlights the complexity of the TIMP1 regulatory network and emphasizes the necessity for multiomics integration to elucidate its pleiotropic effects (26). Notably, while TIMP1 knockdown markedly reduced erastin-induced Fe^2+^ accumulation, its overexpression did not proportionally amplify Fe^2+^ levels, suggesting saturation of TIMP1-dependent iron-regulatory pathways or compensatory feedback mechanisms. This underscores the complexity of iron homeostasis, where TIMP1 is necessary but not solely sufficient to drive pathological iron accumulation. Although our findings suggest that the regulation of ferroptosis by TIMP1 contributes to its antimetastatic effects, additional studies are needed to disentangle ferroptosis-dependent and -independent mechanisms. For example, rescuing ferroptosis (*e.g.*, *via* ferrostatin-1 or GPX4 overexpression) in TIMP1-high cells could determine whether restored viability directly enhances migration.

A limitation of this study is the absence of formal isotype controls for IHC. However, the concordance between IHC and Western blot results, alongside functional ferroptosis assays, supports the specificity of our findings. Future studies will include comprehensive antibody validation.

The dual role of TIMP1 in PCa migration—acting as both a metastasis suppressor and a promoter—reflects its context-dependent interactions with MMPs, cytokine networks, and redox pathways. In early stage tumors, MMP-inhibitory function of TIMP1 likely restrains ECM degradation and invasion (25, 38). However, in advanced or therapy-resistant tumors, cytokine-like signaling of TIMP1 *via* CD63/β1-integrin may dominate, activating prosurvival pathways (*e.g.*, PI3K/AKT) that counteract its antimetastatic effects (9, 10). Our findings further demonstrate that TIMP1 regulates ferroptosis, creating a dynamic balance: TIMP1-overexpressing cells succumb to lipid peroxidation, reducing metastatic potential, whereas TIMP1-low cells evade ferroptosis, favoring survival in oxidative environments (Fig. 5). This interplay underscores the need to stratify TIMP1-targeted therapies based on tumor stage, metabolic state, and TIMP1 expression levels.

Clinically, TIMP1 plays dual roles—as a metastasis suppressor (*via* MMP inhibition) (29, 39, 40, 41) and a promoter of therapy resistance (*via* cytokine signaling) (9, 10). The findings of this study introduce the concept of ferroptosis regulation as a third axis, suggesting that TIMP1 overexpression sensitizes TIMP1-low tumors to oxidative damage, whereas TIMP1 inhibition might counteract ferroptosis resistance in TIMP1-high malignancies. This finding is consistent with studies in colorectal cancer, where TIMP1 knockdown sensitized cells to sorafenib-induced ferroptosis (25, 26, 29), and breast cancer, where TIMP1 expression correlated with chemoresistance (42, 43). Strategies that stratify patients based on TIMP1 expression, guided by biomarkers, have the potential to optimize therapeutic outcomes.

In conclusion, our work positions TIMP1 as a pivotal regulator of ferroptosis in PCa, with implications for cancers marked by oxidative stress. Future research should delineate the crosstalk of TIMP1 with pathways like Nrf2/GPX4 (32) and validate TIMP1-targeted therapies in preclinical models. Furthermore, the combination of TIMP1 modulation with FINs (*e.g.*, erastin) (23, 24) or immunotherapies may enhance efficacy in advanced PCa. The present study demonstrates that by bridging the roles of TIMP1 in metastasis and cell death, new avenues for precision oncology are opened up.

## Experimental procedures

### Cells and culture

Human PCa cell line PC-3M-2B4 cells were purchased from Cell Resource Center, Institute of Basic Medical Sciences, and Chinese Academy of Medical Sciences/Peking Union Medical College. PC-3M-2B4 cells have limited metastatic potential in nude mice. Cells were cultured in RPMI1640 medium (Gibco) supplemented with 10% heat-inactivated fetal bovine serum (Gibco), 100 IU/ml penicillin G (Beyotime), and 100 μg/ml streptomycin (Beyotime) at 37°C, 5% CO_2_, and full humidity. The FIN, 10 μM erastin (Beyotime), and inhibitor, 5 μM ferrostatin-1 (Selleck), were added to the culture medium where indicated. Stock solutions (10 mM erastin and 5 mM ferrostatin-1) were diluted in dimethyl sulfoxide and further in medium to achieve final working concentrations.

### Transfection

PC-3M-2B4 cells were seeded in 24-well plates at a density of 1 × 10^4^ cells per well and transfected at 40%∼60% confluence, incubated for 24 h, and 0.5 ml complete medium added for 48 h incubation. PC-3M-2B4-TIMP1 cells overexpressing TIMP1 were transfected with a pHBLV-CMV-MCS-EF1-ZsGreen-T2A-fLUC vector (Shanghai Hanheng Biological Co, Ltd), and cells were identified by flow cytometry. PC-3M-2B4-shTIMP1 cells with TIMP1 knockout were transfected with a pHBLV-U6-Luc-Puro vector (Shanghai Hanheng Biological Co, Ltd), containing the TIMP1 shRNA sequence: GCAAACTGCAGAGTGGCACTCATTG and selected using puromycin. shRNA sequences were top strand: GATCCGCAAACTGCAGAGAGTGGCACTCATTGTTCAAGAGACAATGAGTGCCACTCTGCAGTTTGCTTTTTTC and bottom strand: AATTGAAAAAAGCAAACTGCAGAGTGGCACTCATTGTCTCTTGAACAATGAGTGCCACTCTGCAGTTTGCG. TIMP1 expression was assessed by Western blotting and quantitative PCR. Primer sequences were: Lv-TIMP1-E/B-F: agaggatctatttccggtgaattcGCCACCATGGCCCCCTTTGAGCCCCCCT; Lv-TIMP1-E/B-r:TTG-TCATCGTCATCCTTGTAGTCGGCTATCTGGGGACCGCAGGGA; Lv-TIMP1-E/B-f:TCCCTGCGGTCCCAGATAGCCGACTACAAGGATGACGATGACAA; and Lv-TIMP1-E/B-R:atccttactagtatcgatggatccTTATTTGTCGTCATCATCCTTATAG (Table S1).

### CCK-8 assay

Cells were inoculated into 96-well plates at 2000 cells per well and counted at 0, 24, 48, and 72 h after attachment. CCK-8 (10 μl; Beyotime) was added to each well, and the cells were incubated at 37 °C, 5% CO_2_ for 1 h. The ELISA instrument was used to measure the absorbance at 450 nm, and a cell growth curve was generated. Experiments were performed in triplicate.

### Wound healing assay

Cells were inoculated into 6-well plates at a density of 3 × 10^4^ per well and cultured at 37 °C, 5% CO_2_ for 24 h before a vertical scratch was made in the cell monolayer using a 1 ml pipette tip. Plates were rinsed three times with sterile PBS, serum-free medium was added, plates were incubated at 37 °C, 5% CO_2_, and photographed at 0, 24, 48, and 72 h. Wound healing was assessed by ImageJ software (National Institutes of Health).

### Cell migration

Cells in the logarithmic phase were incubated with serum-free medium for 24 h. A 200 μl aliquot of 5 × 10^5^ cells/ml in serum-free RPMI1640 medium was added to the upper Transwell chamber, and RPMI1640 medium with 10% fetal bovine serum was added to the lower chamber for 48 h incubation. Medium and cells were removed from the upper chamber, and cells in the lower chamber were fixed with 4% paraformaldehyde at 4 °C for 20 min, stained with 1% crystal violet solution for 10 min, rinsed with PBS, and air-dried. Stained cells were inspected by 200× inverted microscope, photographed, and those in 10 randomly selected fields of view counted.

### Cell invasion

Matrigel (60 μl; Corning), precooled to 4 °C and diluted 1:8 in basal medium, was added to the upper Transwell chamber and incubated at 37 °C and 5% CO_2_ for 3 h to allow polymerization. Excess liquid was removed, 100 μl basal medium was added to the upper chamber, and the protocol for the cell migration assay was repeated.

### Clonal formation

Seven hundred cells/well were inoculated into a 6-well plate, which was incubated at 37 °C and 5% CO_2_ until most clones were observed to have more than 50 cells. Wells were washed three times with PBS, fixed with precooled 4% paraformaldehyde for 30 to 60 min, washed three times with PBS, and stained with 0.1% crystal violet for 10 to 20 min. Cells were rinsed, air-dried, and photographed.

### Subcutaneous tumor formation

Logarithmic phase cells were harvested with 1% trypsin, rinsed with precooled PBS, and resuspended at 2 × 10^7^ cells/ml. A 0.1 ml aliquot of a 1:1 mixture of cell:Matrigel was injected subcutaneously into the upper limbs of 4- to 6-week-old, 16 to 20 g male BALB/C nude mice. Fluorescence intensity was monitored every 2 weeks by In Vivo Imaging System (IVIS) for Small Animals (PerkinElmer IVIS Lumina).

### Subperitoneal tumor transplantation

Humanized PCa tissue was grown subcutaneously, excised, and minced into pieces *<*1 cm^3^. A small incision was made in the renal peritoneum of nude mice with pointed forceps, tumor tissue coated with Matrigel tucked under the renal peritoneum, and the incision closed layer by layer. Biofluorescence intensity was measured every 2 weeks using IVIS. Mice were sacrificed, and tissues were dissected for analysis.

### Small animal *in vivo* imaging

IVIS was performed on nude mice, which had been respiratory anesthetized and injected intraperitoneally with the luciferase substrate, d-luciferin (150 mg/kg in μl). Tumor growth and metastasis were assessed by IVIS 10 min later.

### MDA detection

Tissues or cell lysates were centrifuged at 12,000 rpm for 15 min at 4 °C, and 200 μl supernatant was added to 0.2 ml MDA assay solution (Beyotime) before the absorbance was measured at 532 nm using an enzyme marker. MDA content was calculated from a standard curve and expressed as μM/mg protein.

### Intracellular Fe^2+^ concentration detection

Cells were inoculated at 2000 per well into 96-well plates and incubated overnight at 37 °C in 5% CO_2_. Cells were treated with drugs and incubated for a further 24 h. The supernatant was removed, and the cells were washed three times with serum-free medium. The fluorescence intensity (excitation: 543 nm/emission: 580 nm) of each sample was assessed by multifunctional ELISA with the addition of 1 M ferro-orange solution (Beyotime) and incubation at 37 °C, 5% CO_2_ for 30 min.

### GSH–GSSG detection

About 0, 5, and 10 μl of blank controls, standard curve, and samples were added to a 96-well late with 0, 5, and 10 μl protein removal reagent M and 150 μl total glutathione assay working solution (Beyotime), incubated at 25 °C for 5 min, and 50 μl 0.5 mg/ml NADPH added. Absorbance at 412 nm was measured at time 0- and 5-min intervals or in real time by zymography. Levels of GSSG were estimated from a standard curve.

### GPX4 detection

Cell or tissue lysates were centrifuged at 12,000*g* for 15 min at 4 °C, and supernatants were used. Samples were prepared as follows: control: 183 μl Glutathione Peroxidase Assay Buffer (Beyotime), 5 μl 10 mM GSH solution, and 12 μl 15 mM Peroxide Reagent solution; sample: 178 μl Glutathione Peroxidase Assay Buffer, 5 μl 10 mM GSH solution, 5 μl sample, and 12 μl 15 mM Peroxide Reagent solution, and incubated at 25 °C for 30 min before addition of 6.6 μl 5,5'-dithiobis-(2-nitrobenzoic acid) solution and incubation at 25 °C for 10 min for absorbance at 412 nm measurement.

### H&E stain

The sections were deparaffinized in xylene, hydrated in graded concentrations of ethanol, stained with hematoxylin (Beyotime), differentiated with 1% hydrochloric acid in alcohol, rinsed with tap water to counter-blue, stained with eosin stain (Beyotime), dehydrated in graded concentrations of ethanol, hyalinized in xylene, and sealed in neutral resin. Sections were examined under the microscope after drying. The intensity of the staining was evaluated by a pathologist.

### IHC analysis

Sections were deparaffinized in xylene, rehydrated through a gradient of ethanol, 0.01 M citrate buffer, retrieved with antigen in a microwave oven, quenched with 0.03% H_2_O_2_, washed with PBS, blocked, incubated with primary antibody, washed with PBS, incubated with secondary antibody, reacted with hematoxylin in DAB chromogenic buffer (Maixin Biotechnology), dehydrated through a gradient of ethanol, cleared with xylene, and blocked with neutral resin. Antigen retrieval was performed in an antigen retrieval masking solution. Sections were blocked with blocking solution to seal the antigen, incubated with primary antibody overnight at 4 °C, and then incubated with secondary antibody for 1 h. The intensity of nuclear staining was assessed by one pathologist and scored in a blinded manner, defined as negative (or present in <5% of nuclei), weak, moderate, or strong. Antibodies for IHC (GPX4, TFRC, TF, GCLC, and GCLM) were selected based on commercial validation and consistency with Western blot results (Fig. 5*F*). While isotype controls were not included in this study, adjacent nontumor tissue served as an internal negative reference. Full validation will be provided in subsequent work.

### Parallel reaction monitoring

Peptides from cell samples were subjected to nanospray ionization source and tandem mass spectrometry (MS/MS) in a Q ExactiveTM Plus (Thermo) connected to UPLC. An electrospray voltage of 2.0 kV was applied with an *m/z* scan range of 350 to 1000 for a full scan. The automatic gain control was set at 3 million ions for full MS and 100,000 ions for MS/MS. The maximum ion injection time was set to 20 ms for full MS and automatic for MS/MS. The variable modification is set to carbamidomethyl for cysteine residues and oxidation for methionine residues. Ion types were b, y, and p. Product ions are set from ion 3 to the last ion, with an ion match tolerance of 0.02 Da.

### Protein‒protein interaction network

A search was performed using the STRING database, version 11.0 to identify database entries or sequences associated with differentially expressed proteins with a confidence score of 0.7 or higher. Proteins were visualized using R package network D3.

### Statistical analysis

GraphPad Prism 9 (GraphPad Software, Inc) was used for all statistical analyses. Normally distributed pairs of data were compared by Student’s *t* test, and multiple comparisons were made by one-way or two-way ANOVA. Results are expressed as ¯x¯ ± SD (∗*p <* 0.05, ∗∗*p <* 0.01, ∗∗∗*p <* 0.001, and ∗∗∗∗*p <* 0.0001).

## Data availability

The data that support the findings of this study are available in the methods and supporting information of this article.

## Supporting information

This article contains supporting information.

## Institutional review board statement

All animal experiments were conducted in accordance with the Basel Declaration, and ethical approval was granted by the Animal Care and Use Committee, National Center for Toxicological Evaluation of Fertility Regulating Drugs, Shanghai Institute of Biomedical Technology (IACUC-20220802-01).

## Conflict of interest

The authors declare that they have no conflicts of interest with the contents of this article.
