# Supplementary material for: Tissue inhibitor of metalloproteinase 1 promotes ferroptosis and suppresses prostate cancer metastasis
Source: J Biol Chem. 2025 Apr 2;301(5):108473. doi: 10.1016/j.jbc.2025.108473 (PMC12059338; doi:10.1016/j.jbc.2025.108473)
Supplement: Supplementary data [file mmc1.docx]

**Supplementary**

Three cell samples were subjected to STR labeling for identification using the ExPASy (Expert Protein Analysis System) human cell line. The similarity matching results with the PC-3 cell line are as follows: PC-3M-2B4-shTIMP1 cells showed a 96.00% match, PC-3M-2B4-TIMP1 cells exhibited a 96.00% match, and PC-3M-2B4 cells demonstrated a 96.00% match. The identification work was conducted by Suzhou Ganda Biotechnology Co., and detailed results can be found in Table S1.

***Table S1. Identification results of PC-3M-2B4-shTIMP1, PC-3M-2B4-TIMP1 and PC-3M-2B4 cells analyzed by the STR method***

| **Target Loci** | **Test Results for Submitted Sample** | | | | | | **ExPASy Reference Database Profile** | |
| --- | --- | --- | --- | --- | --- | --- | --- | --- |
|  | **PC-3M-2B4-shTIMP1** | | **PC-3M-2B4-TIMP1** | | **PC-3M-2B4** | | **Database Profile: PC-3** | |
| Amelogenin | X |  | X |  | X |  | X |  |
| D5S818 | 13 |  | 13 |  | 13 |  | 13 |  |
| TPOX | 8 | 9 | 8 | 9 | 8 | 9 | 8 | 9 |
| CSF1PO | 11 |  | 11 |  | 11 |  | 11 |  |
| TH01 | 6 | 7 | 6 | 7 | 6 | 7 | 6 | 7 |
| vWA | 17 |  | 17 |  | 17 |  | 17 |  |
| D7S820 | 8 | 11 | 8 | 11 | 8 | 11 | 8 | 11 |
| D16S539 | 11 | 12 | 11 | 12 | 11 | 12 | 11 |  |
| D13S317 | 11 |  | 11 |  | 11 |  | 11 |  |
| Alleles Match | 96% | | 96% | | 96% | |  | |

Note: 1. The allele matching algorithm only compares the 8 core loci and amylogenin to verify cell identity. Allelic testing of all STR loci is not published to protect donor identity.2. Cell lines with *≥*80% match is considered related. Cell lines with a match between 55% and 80% require further analysis to verify relatedness.***Table S2. Tumorigenic results in PC-3M-2B4-TIMP1, PC-3M-2B4-shTIMP1 and PC-3M-2B4 nude mice***

| **Groups** | **Number of nude mice** | **IVIS Number of detections** | **detection rate(%)** | **Number of IVIS-detected transfers** | **IVIS signal shift detection rate(%)** | **Number of confirmed transfers** | **Tumor metastasis detection rate(%)** |
| --- | --- | --- | --- | --- | --- | --- | --- |
| PC-3M-2B4 | 10 | 9^#^ | 90.00^#^ | —— | —— | 3 | 33.33 |
| PC-3M-2B4-TIMP1 | 10 | 6 | 60.00 | 1 | 16.67 | 1 | 16.67 |
| PC-3M-2B4-shTIMP1 | 10 | 9 | 90.00 | 5 | 55.56 | 5 | 55.56 |
| total | 36 | 15 | —— | 6 | —— | 9 | —— |

Note: Detection rate is the ratio of the number of biofluorescent signals detected by IVIS to the number of surviving animals; IVIS signal metastasis detection rate (%) is the ratio of the number of animals with metastasis detected to the number of animals with tumors detected; Tumor metastasis detection rate (%) is the ratio of the number of experimental animals with metastasis confirmed by both IVIS and HE staining to the number of experimental animals with tumors. #: PC-3M-2B4 cells were not labeled with luciferase and the value is derived from anatomical pathological examination.
